# GTP-Binding-Defective ARL4D Alters Mitochondrial Morphology and Membrane Potential

**DOI:** 10.1371/journal.pone.0043552

**Published:** 2012-08-21

**Authors:** Chun-Chun Li, Tsung-Sheng Wu, Chun-Fang Huang, Li-Ting Jang, Yu-Tsan Liu, Shu-Ting You, Gunn-Guang Liou, Fang-Jen S. Lee

**Affiliations:** 1 Institute of Molecular Medicine, College of Medicine, National Taiwan University, Taipei, Taiwan; 2 Department of Medical Research, National Taiwan University Hospital, Taipei, Taiwan; 3 Division of Molecular and Genomic Medicine, National Health Research Institutes, Miaoli, Taiwan; Karolinska Institutet, Sweden

## Abstract

ARL4D, ARL4A, and ARL4C are closely related members of the ADP-ribosylation factor/ARF-like protein (ARF/ARL) family of GTPases. All three ARL4 proteins contain nuclear localization signals (NLSs) at their C-termini and are primarily found at the plasma membrane, but they are also present in the nucleus and cytoplasm. ARF function and localization depends on their controlled binding and hydrolysis of GTP. Here we show that GTP-binding-defective ARL4D is targeted to the mitochondria, where it affects mitochondrial morphology and function. We found that a portion of endogenous ARL4D and the GTP-binding-defective ARL4D mutant ARL4D(T35N) reside in the mitochondria. The N-terminal myristoylation of ARL4D(T35N) was required for its localization to mitochondria. The localization of ARL4D(T35N) to the mitochondria reduced the mitochondrial membrane potential (ΔΨm) and caused mitochondrial fragmentation. Furthermore, the C-terminal NLS region of ARL4D(T35N) was required for its effect on the mitochondria. This study is the first to demonstrate that the dysfunctional GTP-binding-defective ARL4D is targeted to mitochondria, where it subsequently alters mitochondrial morphology and membrane potential.

## Introduction

ADP-ribosylation factors (ARFs), members of the Ras family of small GTPases, are involved in membrane transport, the maintenance of organelle integrity, membrane lipid modification, and cytoskeletal dynamics [1], [2]. The ARF family members are divided into ARF, ARF-like (ARL), and Sar proteins [1]–[4] based on biochemical activities and sequence similarity. To date, at least six ARFs (five human ARFs), which have >60% sequence identity, and more than 20 ARL proteins, which are 40–60% sequence identical to ARFs or to each other, have been identified [2]. Similar to other GTP-binding proteins, ARF depends on the binding and hydrolysis of GTP, which is controlled by their interaction with specific guanine nucleotide exchange factors (GEFs) and GTPase-activating proteins (GAPs) [2], [3]. The conformational changes that accompany GDP or GTP binding to ARFs are thought to change the affinity of the GTPase for proteins, lipids and membranes [5], [6]. The membrane binding of ARF and most ARL proteins is mediated by both an N-terminal myristoyl group and an N-terminal amphipathic helix. The exposure of the covalently attached myristate and N-terminal amphipathic helix upon GTP binding causes the GTP-bound form of the ARF protein to interact with the lipid bilayer [5].

Three isoforms of ARL4 (i.e., ARL4A, ARL4C, and ARL4D) can be distinguished from the other members of the ARF family by a short basic extension at the C terminus and a short insertion in the loop between the two switch regions [5]. The expression of the ARL4 proteins is developmentally regulated, tissue specific, and dependent on the stage of differentiation [1]–[4]. The unique basic extension at the C terminus of the ARL4 proteins interacts with importin-α and functions as a nuclear localization signal (NLS) to mediate the nuclear translocation of the ARL4s [7]–[9]. ARL4D is also known to interact with heterochromatin protein 1α (HP1α) although the functional relationship between these two proteins remains unknown [8]. ARL4D and ARL4A recruit cytohesin/ARNO to the plasma membrane [10], [11], thereby promoting ARF6 activation and modulating the reorganization of the actin cytoskeletal [10]. ARL4A was recently reported to form complexes with ELMO to promote actin cytoskeleton remodeling and to act with GCC185 to modulate Golgi apparatus organization [12], [13].

A recent study has shown that modifying either terminus of Arf1 through the fusion of a peptide or protein interferes with some, but not all, Arf1 activities and functions [14]. Fusing the C-terminus of ARF6 to GFP also decreased ARF6 membrane association [15]. ARL4D, ARL4A and ARL4C each have an N-terminal myristoylation site and a C-terminal nuclear localization signal (NLS); thus, epitope tags at either end might alter the conformation, localization and function of these proteins. We have previously shown that untagged recombinant ARL4D, which is similar to endogenous ARL4D, is located primarily at the plasma membrane, but can also be detected in the nucleus and cytoplasm. ARL4D(Q80L), a mutant ARL4D protein that mimics GTP-bound ARL4D, exhibited a localization pattern similar to that of the wild-type protein. Interestingly, the GTP-binding-defective mutant ARL4D(T35N) localized to small, punctate structures throughout the cell. The nature of these structures the mechanism by which ARL4D(T35N) is targeted to these structures were not known.

In this study, we report that the GTP-binding-defective mutant ARL4D(T35N) localizes to mitochondria and subsequently alters the mitochondrial morphology and membrane potential. We show that a portion of the endogenous ARL4D localizes to mitochondria. N-terminal myristoylation is required for ARL4D(T35N) mitochondrial targeting. The association of ARL4D(T35N) with the mitochondria reduced mitochondrial membrane potential and caused mitochondria fragmentation, but there was no evidence suggesting that ARL4D(T35N) affects cell viability or proliferation. Removing the C-terminal NLS region facilitated the mitochondrial association of ARL4D, but reduced the effects of ARL4D(T35N) on mitochondrial membrane potential. We infer that GTP-unbound ARL4D is targeted to the mitochondria to alter mitochondrial function via its C-terminal NLS region.

## Results

### GTP-binding-defective ARL4D(T35N) Localizes to Mitochondria

**Figure 1:**
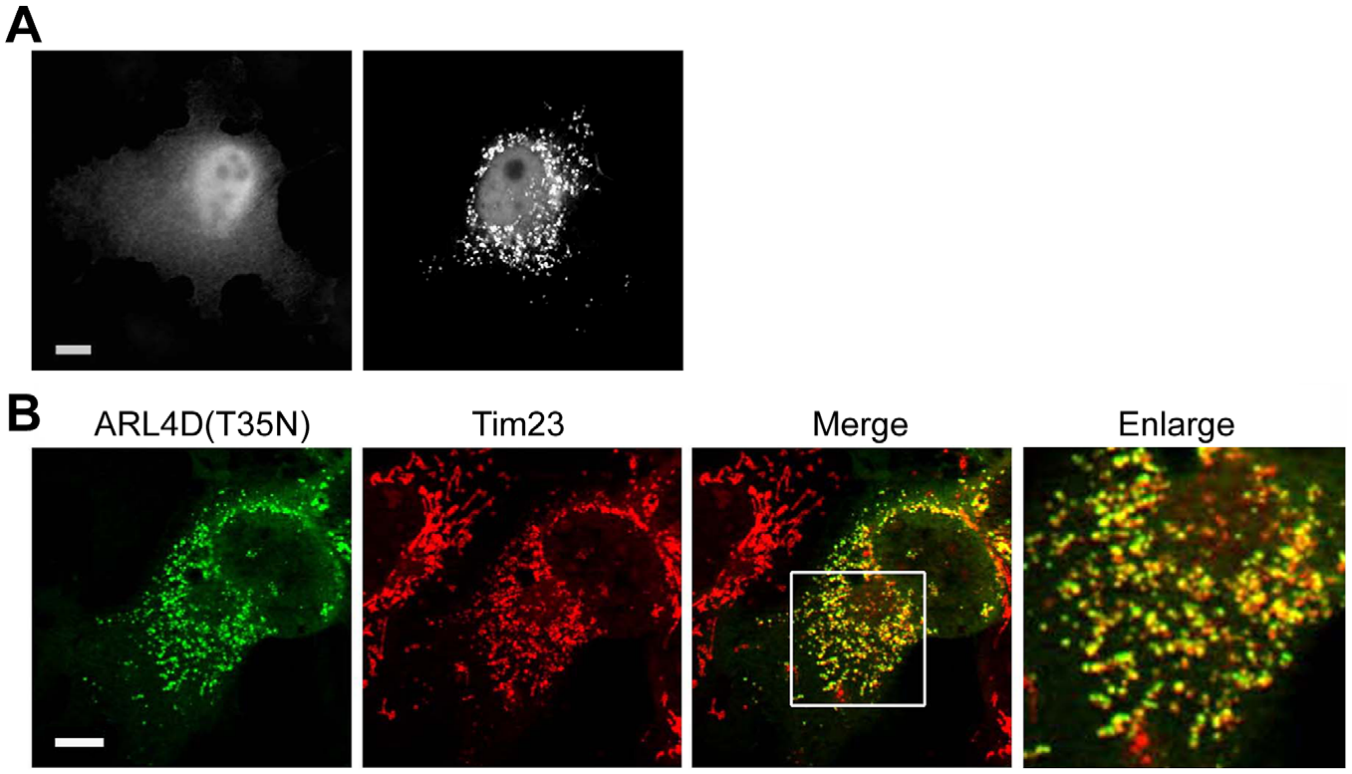
Detection of ARL4D(T35N) in the mitochondria. (A) COS-7 cells were fixed 36 h after transfection, stained with anti-ARL4D antibody, and analyzed by fluorescence microscopy. ARL4D(T35N) displayed a heterogeneous distribution. Two representative images illustrating the cellular distribution of ARL4D(T35N) are shown. In ∼50–60% of the cells, ARL4D(T35N) was observed in punctuate collections throughout the cells (right panel). (B) COS-7 cells were transiently transfected with plasmids encoding ARL4D(T35N), fixed, and stained with antibodies against ARL4D and Tim23 before confocal microscopy. The right panel shows an enlarged view of the boxed area. ARL4D(T35N) colocalized with mitochondria, which exhibited a small and fragmented fission-like morphology. Bars, 10 µm.

Both our group and Katayama et al. have previously reported that overexpressed wild-type (WT) and the putative GTP-bound ARL4D mutant (Q80L) localize to the plasma membrane, while the GTP-binding-defective mutant ARL4D(T35N) locates to perinuclear punctate structures [10], [16]. However, the detailed subcellular localization and function of ARL4D(T35N) remains unclear. To better understand the function of ARL4D, we first analyzed the intracellular localization of overexpressed ARL4D(T35N) in COS cells by confocal fluorescence microscopy (Figure 1). ARL4D(T35N) displayed two different distribution patterns. In ∼50–60% of the untagged ARL4D(T35N)-expressing cells, ARL4D(T35N) was observed in the nuclei and in punctate structures scattered throughout the cytoplasm that were clustered in the perinuclear region, as previously reported (Figure 1A, right panel) [10], [16]. In cells that did not exhibit this pattern, the overexpressed ARL4D(T35N) was diffusely distributed throughout the cytoplasm (Figure 1A, left panel). The perinuclear punctate structures containing ARL4D(T35N) colocalized with the mitochondrial marker, Tim23 (Figure 1B). The colocalization of ARL4D(T35N) and β-COP (cis-Golgi marker), calnexin (ER marker), EEA1 (early endosome marker), transferrin (recycling endosome marker), mannose 6-phosphate receptor (a protein cycling between the TGN and endosomes), or Lamp-1 (lysosome marker) was limited (Figure S1). An apparent colocalization between ARL4D(T35N) and Tim23 was also observed in other cell lines, such as A431, HeLa, and RD cells, indicating that the mitochondrial localization of ARL4D(T35N) is not cell type-specific (data not shown). We also noted that the mitochondrial morphology was different between the ARL4D(T35N) expressing cells and the neighboring non-transfected cells. The mitochondria of the cells overexpressing ARL4D(T35N) were small and fragmented, in contrast with the tubular networks observed in the neighboring cells (Figure 1B, enlarged). This result suggests that the expression of ARL4D(T35N) might affect the dynamics of mitochondrial fission/fusion [17], [18].

**Figure 2:**
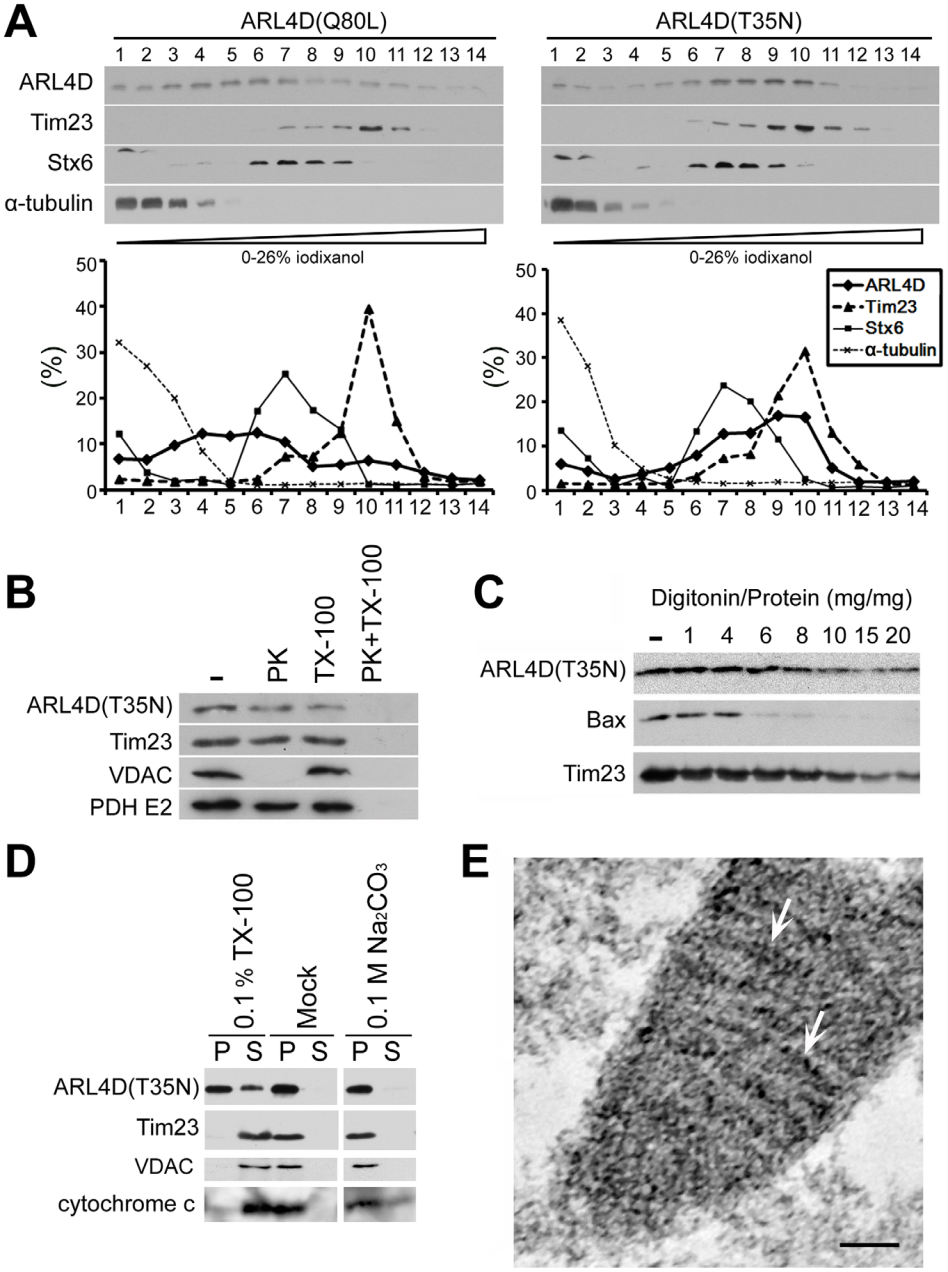
ARL4D(T35N) localized to the mitochondrial inner membrane. (A) The fractionation of ARL4D(Q80L) or ARL4D(T35N)-expressing cells by gradient centrifugation. The postnuclear supernatants of COS-7 cells expressing ARL4D(Q80L) or ARL4D(T35N) obtained at 48 h after transfection were fractionated on iodixanol gradients as described in the Materials and Methods. Samples collected from the top of each indicated fraction were analyzed by Western blot with antibodies against ARL4D, Tim23, syntaxin-6 (Stx6), and α-tubulin. The protein levels in each fraction are reported as the percentage of total protein recovered, as determined by densitometry. (B) The mitochondria-enriched membrane fractions of COS cells expressing un-tagged ARL4D(T35N) were incubated with or without (−) 50 µg/ml proteinase K (PK) in the presence or absence of 0.1% Triton X-100 (TX-100). The samples were precipitated with TCA and analyzed by Western blotting with antibodies against ARL4D, Tim23, VDAC, and pyruvate dehydrogenase (PDH)-E2. (C) The mitochondria-enriched membrane fractions from COS cells expressing untagged ARL4D(T35N) were treated with different digitonin/protein ratios, varying from 1 to 20, as described in the Materials and Methods. The proteins present in the membrane fractions after digitonin treatment were analyzed by Western blotting. (D) The mitochondria isolated from COS cells expressing untagged ARL4D(T35N) were treated with 0.1% Triton X-100, 0.1 M Na_2_CO_3_ (pH 12) or buffer (Mock) and centrifuged to separate the soluble supernatants (S) and membrane pellets (P). The samples were analyzed by Western blotting with antibodies against the indicated proteins. (E) Cryosections of COS-7 cells that had been transfected with the plasmid encoding ARL4D(T35N)-myc were processed for immunogold EM to detect ARL4D(T35N)-myc. Gold particles indicating the presence of ARL4D(T35N) (gold particles, arrows) decorated the mitochondrial inner membrane. Bar, 100 nm.

The subcellular distribution of exogenous ARL4D(T35N) and ARL4D(Q80L) was also determined by the density gradient centrifugation of postnuclear supernatants (Figure 2A). Different distribution profiles were observed for ARL4D(T35N) and ARL4D(Q80L). ARL4D(T35N) was distributed in the heavier fractions (8–11) along with Tim23, whereas ARL4D(Q80L), which localizes to the plasma membrane [10], [11], was mainly found in the lighter fractions (3–7). The similar fractionation profiles of ARL4D(T35N) and Tim23, coupled with their colocalization as observed using fluorescence confocal microscopy, demonstrate that a portion of ARL4D(T35N) localizes to the mitochondria.

To determine the sub-mitochondrial localization of ARL4D(T35N), we treated mitochondria that were isolated from ARL4D(T35N)-expressing COS-7 cells with proteinase K, a non-specific serine protease that cannot penetrate mitochondria (Figure 2B). The integral mitochondrial outer membrane protein VDAC was digested by proteinase K treatment. In contrast, the mitochondrial inner membrane protein Tim23 and the mitochondrial matrix protein pyruvate dehydrogenase were not digested. We found that a portion of ARL4D(T35N) also remained intact. Solubilizing the mitochondrial membranes with Triton X-100 allowed proteinase K to digest each of these proteins. The resistance to proteinase K treatment indicates that a portion of ARL4D(T35N) resides inside the mitochondria, rather than on the cytosolic surface (Figure 2B).

We next used various concentrations of digitonin to sequentially disrupt the outer and inner mitochondrial membranes to examine the sub-mitochondrial localization of ARL4D(T35N) (Figure 2C). Proteins that are associated with the mitochondrial outer membrane, such as Bax, were solubilized at a low concentration of digitonin, while proteins in the inner mitochondrial membrane such as Tim23, were solubilized only at high concentrations of digitonin. Similar to Tim23, ARL4D(T35N) was only solubilized at the higher concentration of digitonin, indicating that ARL4D(T35N) might associate with the mitochondrial inner membrane or reside in the lumen. We also treated isolated mitochondria with Na_2_CO_3_, which efficiently dissociates peripherally associated, but not integral, membrane proteins (Figure 2D). In contrast to cytochrome c, which is loosely associated with the mitochondrial inner membrane and was found in the supernatant after Na_2_CO_3_ treatment, ARL4D(T35N) remained in the membrane fractions, along with VDAC and Tim23 (Figure 2D). Finally, we used immuno-electron microscopy to directly determine the sub-mitochondrial localization of ARL4D(T35N). C-terminal myc-tagged ARL4D(T35N) was expressed in COS cells, and the localization of the expressed protein was detected using a monoclonal anti-myc antibody. As shown in Figure 2E, the gold grains were enriched within the mitochondria, adjacent to the mitochondrial inner membrane. Taken together, we identified the punctate perinuclear structures that were labeled with GTP-binding-defective ARL4D(T35N) as mitochondria and concluded that a portion of ARL4D(T35N) is associated with the mitochondrial inner membrane. We also analyzed the subcellular localization of the GTP-binding-defective mutants of the other two ARL4 family proteins, ARL4A(T34N) and ARL4C(T27N). Similar to ARL4D(T35N), ARL4C(T27N) was detected at mitochondria, while ARL4A(T34N) showed a diffuse distribution in the cytoplasm (Figure S2A). This result suggests that ARL4D and ARL4C, but not ARL4A, may function at the mitochondria.

The ARL4D(T35N) mutant is homologous to ARF1(T31N), in which the conserved Thr residue in the phosphate-binding P-loop has been replaced by Asn. Initially, it was thought that these mutants remain in constitutively GDP-bound states [19]. However, Macia et al. [20] reported that ARF6(T27N) showed a lowered affinity for both GDP and GTP. These authors further identified ARF6(T44N) as a true GDP-bound, inactive form of ARF6 that has a low affinity for GTP and is mostly GDP-bound in vivo. We therefore constructed an ARL4D(T52N) mutant homologous to ARF6(T44N) and examined its nucleotide-binding by fluorescent N-methylanthraniloyl (mant)–labeled guanine nucleotides-binding assay [21]–[23]. Bacterial-recombinant ARL4D(T52N) and ARL4D(T35N) proteins were prepared and tested for their nucleotide-binding status. As shown in Figure S3, we found that there was an ∼6-fold increase of the mant-GDP emission signal, but no enhancement of the fluorescence of mant-GTP emission when incubated with ARL4D(T52N). However, incubation with ARL4D(T35N) resulted in only an ∼3-fold increase in mant-GDP fluorescence emission and ∼1.3-fold increase in mant-GTP. These results suggest that recombinant ARL4D(T52N) has higher affinity for GDP than does ARL4D(T35N), which is parallel to the nucleotide binding properties of ARF6(T44N) and ARF(T27N). We further examined the subcellular localization of ARL4D(T52N) and found that ARL4D(T52N) was also detected at mitochondria (Figure S2A). All together, our findings indicate that GTP-binding-defective ARL4D can localize to mitochondria.

### A Portion of Endogenous ARL4D Localizes to Mitochondria

**Figure 3:**
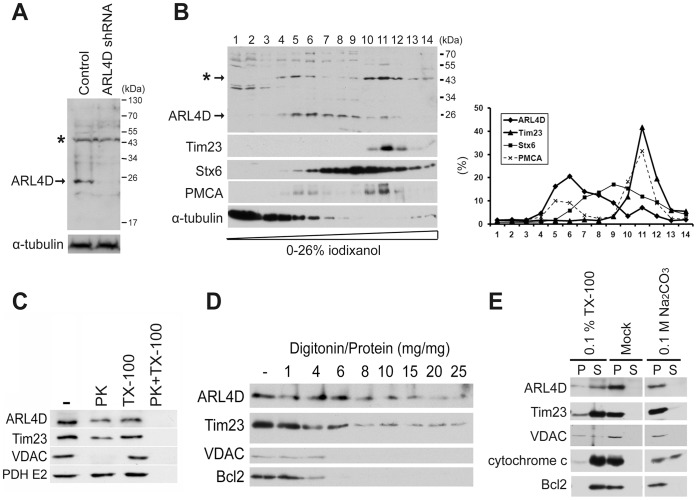
A portion of endogenous ARL4D localized to the mitochondria. (A) ARL4D was knocked down by stably transfecting HeLa cells with a plasmid encoding ARL4D-specific shRNA as described in the Materials and Methods. The efficiency of the ARL4D knockdown was established by immunoblotting with an antibody against ARL4D. α-tubulin was analyzed as an internal control. The asterisk (*) indicates a nonspecific band that cross-reacted with the ARL4D antibody in the total cell lysate. (B) The postnuclear supernatants of HeLa cells were fractionated by iodixanol gradient centrifugation and analyzed by Western blotting. The protein amounts in each fraction are reported as the percentage of total protein recovered, as determined by densitometric quantification. (C) The mitochondria-enriched fractions of HeLa cells were treated and analyzed as described in Figure 2B. (D) The mitochondria-enriched fractions of HeLa cells were treated with different concentrations of digitonin. The proteins present in the membrane fractions after digitonin treatment were analyzed by Western blot. (E) The mitochondria-enriched HeLa cell fractions were treated as described in Figure 2D. The proteins present in the soluble supernatants (S) or membrane pellets (P) were determined by Western blotting.

Endogenous ARL4D is primarily found at the plasma membrane, but it is also present in the nucleus and cytoplasm in HeLa cells [2]. To examine whether endogenous ARL4D could localize to mitochondria, we analyzed postnuclear supernatant obtained from HeLa cells by density gradient centrifugation. To assess the specificity of our ARL4D antibodies, we used HeLa cells that were stably transfected with a plasmid encoding shRNA targeting ARL4D as a control. When we compared the total lysates from the ARL4D-knockdown and control cells, a ∼25-kDa band was detected in the lysate of the control cells only, indicating that this band is the ARL4D protein (Figure 3A). However, because several other bands were clearly visible in both lysates, we assessed the endogenous protein distribution in HeLa cells by iodixanol density gradient fractionation of the postnuclear supernatant. While the majority of the endogenous ARL4D localized to fractions 5–9, some localized to heavier fractions (10–12), and the distribution of ARL4D in the gradient paralleled that of the plasma membrane protein PMCA. The distribution of ARL4D in fractions 10–12 (approximately 10% of total ARL4D) essentially overlapped with the single peak found for the mitochondrial protein Tim23 (Figure 3B). To assess whether endogenous ARL4D localized to mitochondria, we isolated mitochondria for further analysis. We first treated the isolated mitochondria with proteinase K in the presence or absence of Triton X-100. Similar to Tim23 and pyruvate dehydrogenase, a portion of the endogenous ARL4D was resistant to proteinase K digestion in the absence of Triton X-100 (Figure 3C). We next used increasing concentrations of digitonin to sequentially solubilize the mitochondrial membranes. We found that endogenous ARL4D was solubilized at digitonin concentrations similar to those required to solubilize Tim23, but not those that were sufficient to solubilize the mitochondrial outer membrane proteins VDAC or Bcl2 (Figure 3D). We also treated the isolated mitochondria with Na_2_CO_3_ to extract the peripherally associated proteins from the mitochondrial membranes. In contrast to cytochrome c, which is loosely associated with the mitochondrial inner membrane and was extracted to the supernatant after Na_2_CO_3_ treatment, endogenous ARL4D remained in the membrane pellets (Figure 3E). In conclusion, our data showed that a portion of the endogenous ARL4D resides in the mitochondria due to an association with the inner mitochondrial membrane. We failed to confirm that endogenous ARL4D localizes to the mitochondria by fluorescence microscopy because similar mitochondrial staining intensities were observed in control cells and ARL4D-knockdown cells after staining with our ARL4D antibody. Much of this mitochondrial staining may be due to the unidentified ∼50-kDa protein distributed in the mitochondrial fractions that is also recognized by this ARL4D antibody (Figure 3A and 3B, asterisks).

### The Roles of N-terminal Myristoylation and the C-terminal Nuclear Localization Signal in the Localization of ARL4D(T35N) to Mitochondria

**Figure 4:**
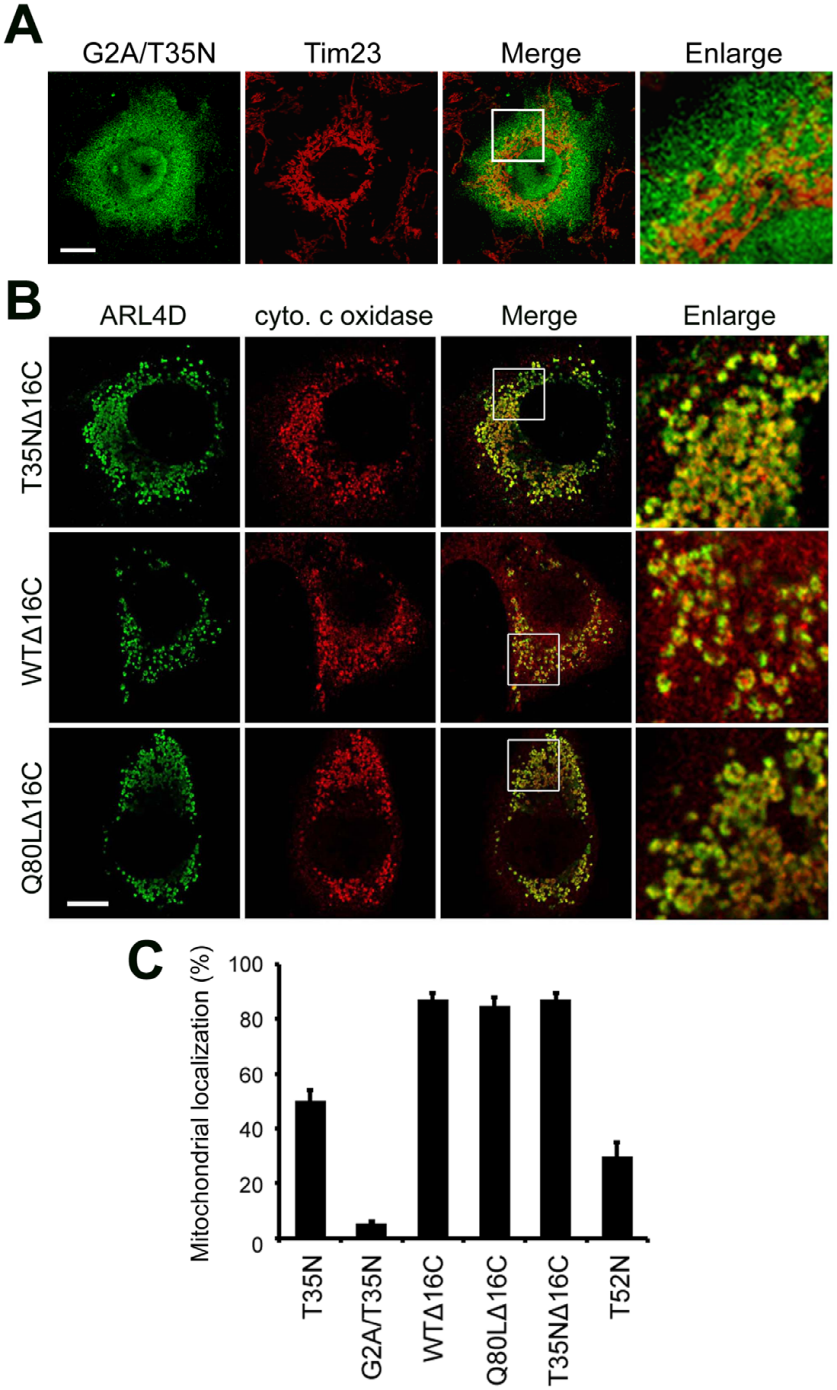
Intracellular localization of ARL4D(G2A/T35N) and ARL4D mutants with a C-terminal deletion. (A) COS cells were transiently transfected with plasmids encoding untagged ARL4D(G2A/T35N). At 36 h after transfection, the cells were stained for ARL4D and Tim23. Bar, 10 µm. (B) COS-7 cells were transfected with plasmids encoding untagged ARL4D mutants with C-terminal deletions of 16 amino acids (T35NΔ16C, WTΔ16C, or Q80LΔ16C), stained with antibodies against ARL4D and cytochrome c oxidase, and examined by confocal microscopy. The magnified figures show that the mitochondria in the ARL4DΔ16C transfected cells exhibit an abnormal, swollen morphology. Bar, 10 µm. (C) Quantification of the ARL4D mutant protein present in the mitochondria. The data represent the results of three independent experiments. At least 100 cells from each population were examined. The data represent the mean ± SD. * p<0.005 vs ARL4D(T35N) transfected cells.

The N-terminal α-helix and myristoyl chain are two key elements that contribute to the membrane association of ARFs [24], [25]. To test whether myristoylation is important for the mitochondrial membrane association of ARL4D(T35N), we replaced the myristoylated amino acid residue Gly2 with Ala. The myristoylation-defective mutant ARL4D(G2A/T35N) localized to the cytosol and did not associate with the mitochondria (Figure 4A and 4C), indicating that N-terminal myristoylation of ARL4D(T35N) is required for its mitochondrial membrane association.

ARL4s contain an NLS extension of ∼13 basic amino acids at their C-termini that is not found in most ARF family proteins [5], [7]. To test whether the C-terminal NLS region plays a role in the mitochondrial targeting of ARL4D(T35N), we examined the localization of an ARL4D protein that lacks the C-terminal NLS region in COS-7 cells. When the 16 C-terminal amino acids were deleted, ARL4D(T35N)Δ16C localized to the mitochondria and was not distributed diffusely in the cytoplasm. Surprisingly, both wild-type ARL4D and the putative GTP-bound ARL4D mutant harboring this 16 amino acid deletion (ARL4DΔ16C and ARL4D(Q80L)Δ16C) also localized to the mitochondria, and these proteins were not observed at the plasma membrane (Figure 4B and 4C). Cells that expressed either of the ARL4D C-terminal truncation constructs showed abnormal, swollen mitochondria (Figure 4B). These data suggest that the C-terminus of ARL4D might prevent GTP-bound ARL4D from associating with mitochondria, in addition to regulating the nuclear-cytoplasmic shuttling of ARL4D.

### ARL4D(T35N) Dissipates the Mitochondrial Membrane Potential through its C-terminus

**Figure 5:**
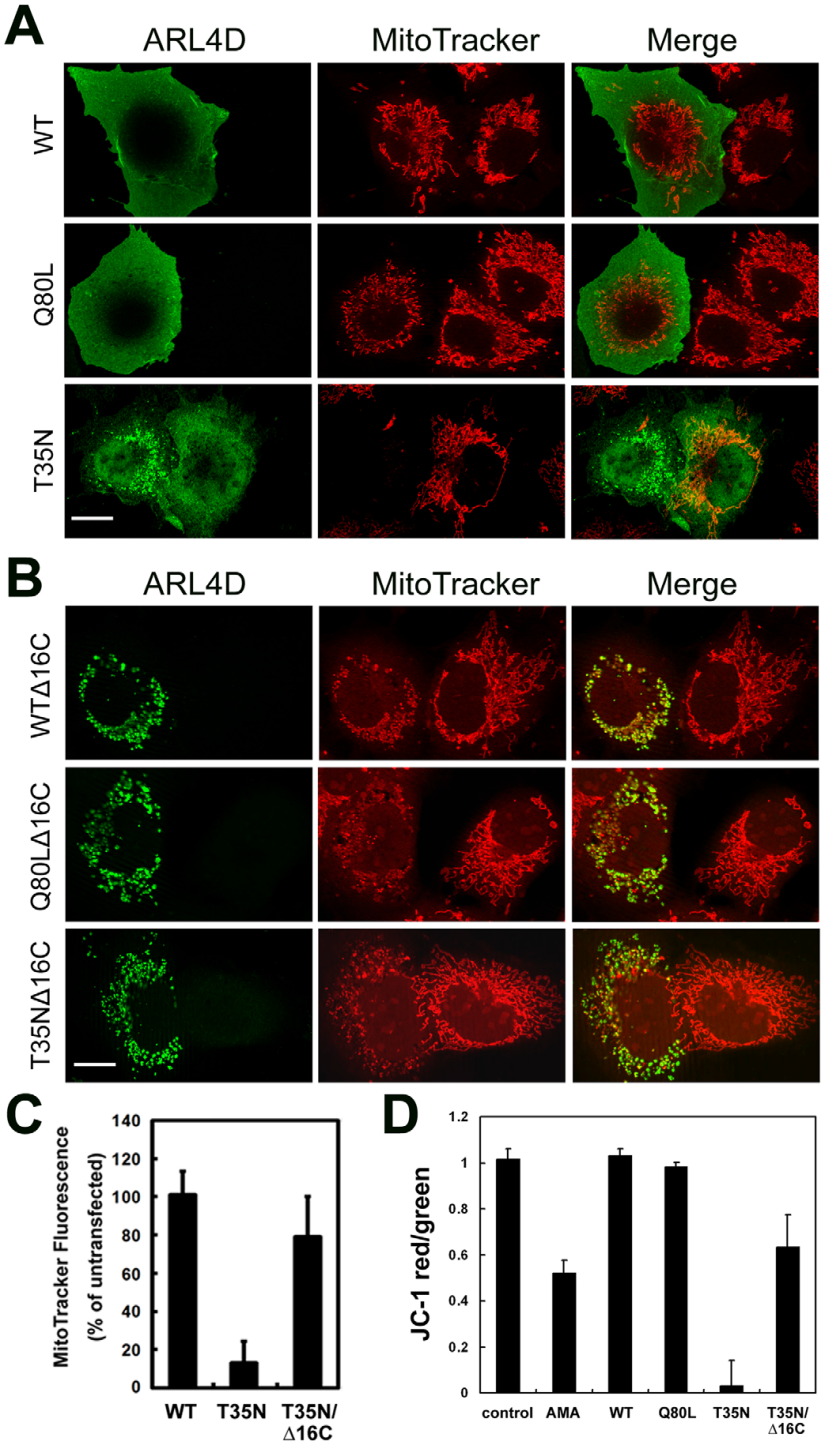
ARL4D(T35N) dissipates the mitochondrial membrane potential. (A and B) COS-7 cells were transiently transfected with plasmids encoding untagged ARL4D and its indicated mutants for 48 h, incubated with MitoTracker Red CMXRos and antibodies against ARL4D, and analyzed by confocal microscopy. Bars, 10 µm. (C) The fluorescence intensity of the MitoTracker-labeled mitochondria in the cells transfected with the ARL4D constructs and in the surrounding untransfected cells was quantified as described in the Materials and Methods. Twenty transfected cells were analyzed for each ARL4D mutant. The data shown represent the means ± SD of the values obtained from three independent experiments. (D) The mitochondrial membrane potential of RD cells transfected with the indicated ARL4D constructs was measured by JC-1 staining as described in the Materials and Methods. The data represent the mean ± SD from three independent experiments.

To study the significance of the ARL4D(T35N) association with mitochondria, mitochondrial function was examined. We first used MitoTracker Red CMXRos, a mitochondrial membrane potential (ΔΨ_m_)-sensitive fluorescent mitochondrial dye, to monitor the ΔΨ_m_ in cells expressing ARL4D mutant proteins. MitoTracker Red CMXRos accumulates only in actively respiring mitochondria that have an intact ΔΨ_m_ [26]. As shown in Figure 5A, cells that overexpressed ARL4D(WT) or ARL4D(Q80L) showed normal MitoTracker Red staining and tubular mitochondria. Two distinct MitoTracker Red staining patterns were found in the ARL4D(T35N)-expressing cells. When ARL4D(T35N) was localized to the mitochondria, MitoTracker Red did not stain the mitochondria. In contrast, when ARL4D(T35N) was diffusely distributed, the MitoTracker Red staining pattern was similar to that observed in control cells. Similarly, when the overexpressed ARL4D(T52N) or ARL4C(T27N) proteins were localized to the mitochondria, MitoTracker Red did not bind to the mitochondrial membranes, indicating that the ΔΨm was reduced in these cells. However, the overexpression of ARL4A(T34N) did not affect MitoTracker Red mitochondrial staining (Figure S2B). These results suggest that the binding of GTP-binding-defective ARL4 family proteins to the mitochondria might affect the ΔΨm. The ARL4D(T35N)-mediated dissipation of the ΔΨm was not affected by the co-expression of Bcl2 or by pre-treatment with cyclosporine A, a mitochondrial permeability transition inhibitor (data not shown), suggesting that the ARL4D(T35N) or ARL4D(T52N)-mediated disruption of ΔΨm may not be caused by mitochondrial membrane permeability transition. Furthermore, the depolarization of mitochondria by treatment with hydrogen peroxide or rotenone did not increase the percentage of transfected cells in which ARL4D(T35N) localized to the mitochondria (data not shown), indicating that the mitochondrial targeting of ARL4D was not caused by mitochondrial depolarization.

Because C-terminally truncated ARL4D(WT), ARL4D(Q80L), and ARL4D(T35N) all localized to the mitochondria, we next measured the ΔΨ_m_ in cells expressing these truncation mutants. Although the MitoTracker Red signal seemed to be lower in cells expressing these mutants than in non-transfected control cells (Figure 5B and 5C), the reduction was not as obvious as the reduction observed in cells with mitochondrial ARL4D(T35N) (Figure 5C). To confirm the dissipation of the ΔΨ_m_ in the ARL4D-expressing cells, we used another ΔΨ_m_-sensitive dye, JC-1, and quantified its intensity by flow cytometry. At a low ΔΨ_m_, JC-1 exists as a green fluorescent monomer; at a high ΔΨ_m_, JC-1 forms red fluorescent “J-aggregates”. Thus, the ratio of red-to-green fluorescence depends on the ΔΨ_m_ but not on the mitochondrial size, shape, or density. We calculated the ΔΨ_m_ of cells expressing ARL4D and its mutants (Figure 5D) by calculating the JC-1 red-to-green ratio as described in the Materials and Methods. Consistent with the results obtained with MitoTracker Red staining, ARL4D(T35N) significantly lowered the JC-1 red-to-green ratio, indicating the dissipation of ΔΨ_m_. Meanwhile, ARL4D(T35N) Δ16C only slightly affected the ΔΨ_m_. The significant difference in MitoTracker Red and JC-1 staining between the cells expressing ARL4D(T35N) and ARL4D(T35N)Δ16C suggests that the C-terminus of ARL4D not only regulates the mitochondrial localization of ARL4D but also contributes to the disruption of the ΔΨ_m_.

### Dissipation of the Mitochondrial Membrane Potential by ARL4D(T35N) does not Cause Apoptosis

**Figure 6:**
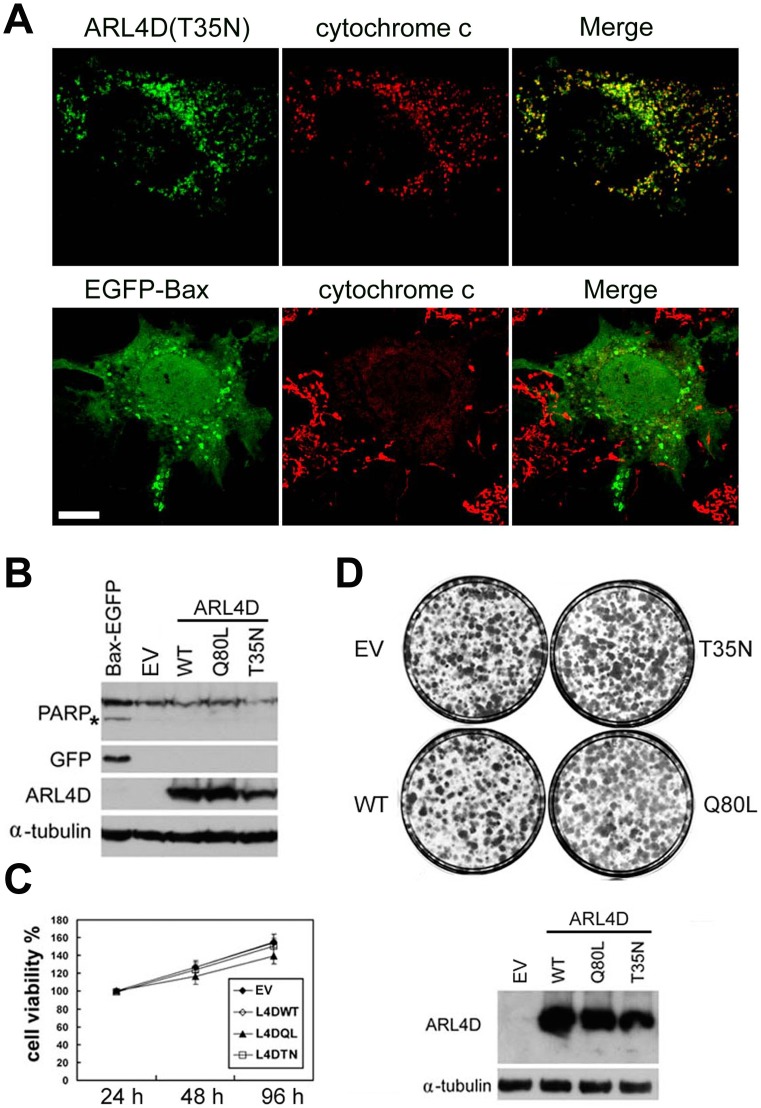
ARL4D(T35N) overexpression does not cause cytochrome c release from mitochondria, induce apoptosis, or affect cell viability. (A) At 36 h hours after transient transfection with plasmids encoding untagged ARL4D(T35N) or Bax-EGFP, COS-7 cells were fixed and incubated with antibodies against ARL4D or GFP plus cytochrome c. Bars, 10 µm. (B) GFP-Bax and the indicated ARL4D constructs were transiently expressed in COS cells. At 48 h after transfection, the cells were harvested, lysed and subjected to Western blot analysis with antibodies against ARL4D, GFP, PARP, or α-tubulin. *, cleaved PARP. (C) The viability of the RD cells was determined by MTT assay at 24, 48 and 96 h after transfection with plasmids encoding ARL4D or the indicated mutants. (D) RD cells were transfected with ARL4D, mutants, or an empty vector (EV). The stably transfected cells were subjected to G418 selection for two weeks, and the colonies remaining on the plates were stained with crystal violet (upper panel). The expression of ARL4D and its mutants after G418 selection was measured by Western blot (lower panel).

The dissipation of the ΔΨm has been shown to initiate cell death signaling, such as the permeability transition signal for apoptosis [27]–[29]. Therefore, we assessed whether the ARL4D(T35N)–induced dissipation of ΔΨm leads to cell death. Cytochrome c release and the cleavage of poly-ADP-ribose-polymerase (PARP), two indicators of apoptosis, were assessed in ARL4D mutant-expressing cells. Consistent with a previous report [30], cytochrome c was released from the mitochondria upon the overexpression of Bax-EGFP. However, the expression of ARL4D(T35N) did not cause cytochrome c release (Figure 6A). PARP is cleaved by certain caspases during apoptosis. The overexpression of Bax-EGFP led to the cleavage of PARP; however, PARP was not cleaved in the cells that expressed ARL4D(T35N) (Figure 6B). These results indicate that the expression of ARL4D(T35N) did not promote apoptosis.

We also assessed the effect of ARL4D(T35N) on cell viability and proliferation. MTT assays were performed on RD cells at 24, 48 and 96 h after transfection with the wild-type or mutant forms of ARL4D. The viability of cells overexpressing ARL4D(T35N) was similar to that of the control cells or cells expressing wild-type or Q80L mutant ARL4D (Figure 6C). To ensure that the duration of ARL4D(T35N) expression was sufficient for the protein to exert its effects, we selected transfected cells with G418 and assessed the colony formation abilities of these ARL4D-expressing cells. After two weeks of selection, we found that ARL4D(T35N) expression did not alter the colony-forming ability of the cells (Figure 6D). In contrast, the colony formation ability of the cells expressing EGFP-Bax was completely abolished (data not shown). Taken together, our results indicate that the ARL4D(T35N)-induced dissipation of ΔΨm did not cause apoptosis or alter cell viability.

## Discussion

The appropriate function of ADP-ribosylation factors depends on the precise temporal and spatial control of their GTP binding and hydrolysis. The conformational differences that accompany the binding of GDP or GTP can directly account for the changes in their affinities for specific proteins, lipids, and membranes [1], [3]. By expressing two ARL4D Thr-to-Asn mutants, T35N and T52N, we found that inactive forms of ARL4D localize to the mitochondria. Thr35 is a conserved residue in the phosphate-binding P-loop, mutation of which has been used to investigate the inactive, presumably GDP-bound, form of small GTP-binding proteins. However, a study by Macia et al. showed that mutation of this conserved Thr residue in ARF6 results in a mutant defective in guanine nucleotide binding with low affinities for GTP and GDP (Macia et al., 2004). These reserachers subsequently cloned a new mutant, ARF6(T44N), based on structural information of the protein. ARF6(T44N) preferentially binds to GDP over GTP and is locked in the GDP-bound conformation. In agreement of these authors’ results, we also found ARL4D(T35N) and (T52N) exhibited different nucleotide binding properties using bacterially expressed recombinant proteins. We found that the ARL4D(T35N) mutant has low affinity for GTP and GDP, while the (T52N) mutant had a high affinity for GDP without apparent GTP binding. These experiments were performed *in vitro* using recombinant proteins. The nucleotide binding and conformational properties of these mutants would be fine-tuned *in vivo* when associated with regulatory proteins. Nevertheless, our findings suggest that ARL4D mutants defective in GTP binding localize to the mitochondria.

It is known that the subcellular localization of ARL4D depends on its guanine nucleotide-binding state, N-terminal myristoylation status, and C-terminal NLS [10], [11]. The mitochondrial localization of ARL4D is also dependent on N-terminal myristoylation and the C-terminal NLS. Interestingly, a portion of ARL2-GTP was also found to localize to the mitochondria [31]. Although ARL2 was found to lack covalent N-terminal myristoylation, it was assumed that the N-terminal amphipathic α-helix of ARL2 serves as a potential mitochondrial import sequence [31]. The N-terminal myristoylation of ARL2 provides a certain baseline affinity for the lipid bilayer of the plasma membrane [10]. The requirement of the N-terminal myristoylation for the localization of GTP-binding-defective ARL4D(T35N) and ARL4D(T52N) to the mitochondria suggests that a baseline affinity for the mitochondrial membrane is required for mitochondrial entry. The mechanisms that communicate with each other to coordinate ARL4D association with different lipid bilayers, such as the plasma membrane or mitochondria, remain to be elucidated.

ARL4 proteins have longer Ras-like interswitches and longer C-terminal regions than do other ARF/ARL proteins [5]. The unique basic extension at the C terminus of ARL4D functions as an NLS that interacts with importin-α to mediate ARL4D’s nuclear import [7], [8]. Deleting 16 amino acids from the C-terminus of ARL4D abrogates ARL4D binding to cytohesin-2/ARNO [10], indicating that this C-terminal region might have additional functions aside from its role as an NLS. We found that WT and Q80L ARL4D mutants that lack the last 16 amino acids localized to the mitochondria in a fashion similar to that of the TN mutants. However, no information about the three-dimensional structure of any member of the ARL4 family is available, and the role that this C-terminal extension plays in the conformation of ARL4D is still unclear. We propose that the specific ARL4D conformation required for mitochondrial targeting might be masked by the C-terminal NLS. After GTP hydrolysis, ARL4D adopts a GDP-bound or empty conformation that enables its recruitment or retention at the mitochondria.

We found that overexpressed ARL4D(T35N) either localizes to fragmented mitochondria or is diffusely distributed in the cytoplasm of COS-7 cells. Longer transfection periods seem to enhance the mitochondrial localization of ARL4D(T35N) (data not shown). In addition to the two GTP-binding defective mutants, we also found that endogenous ARL4D shifted to the heavier density gradient fractions after a longer culture time (24 h versus 72 h) (data not shown). These observations suggest that ARL4D might execute different functions in different organelles, depending on the physiological conditions of the cell.

Our analysis revealed that ARL4D(T35N) and ARL4D(T52N) are tightly associated with the mitochondrial inner membrane. The mitochondrial respiratory chain generates an electrochemical proton gradient, also known as the mitochondrial membrane potential (ΔΨm), which is important for ATP production and mitochondrial protein transport [32]–[34]. Several factors affect mitochondrial membrane potential, including mitochondrial fission and fusion. Mitochondrial depolarization is known to induce mitochondrial fragmentation [35] by inhibiting organelle fusion, while the dynamics of and equilibrium between mitochondrial fission and fusion are essential for maintaining normal ΔΨm and respiration [17], [18]. The tight association between ARL4D(T35N) and ARL4D(T52N) and the mitochondrial membrane not only dissipates the mitochondrial membrane potential but also causes mitochondrial fragmentation, indicating a possible role for ARL4D(T35N) and ARL4D(T52N) in the regulation of mitochondrial dynamics and the respiratory chains.

Mitochondrial permeability transition inhibitors could not rescue these phenotypes, suggesting that the ARL4D(T35N)-or ARL4D(T52N)-induced disruption of ΔΨm may not be caused by a transition in mitochondrial membrane permeability. Other signaling pathways that disrupt the ΔΨm have been shown to lead to mitochondrial swelling, outer membrane rupture, and the release of apoptotic activators such as cytochrome c. Our data show no clear evidence of cytochrome c release in ARL4D(T35N)-expressing cells, and cell viability was not altered in these mutants. Interestingly, we did not observe the same mitochondrial fragmentation or reduction in membrane potential in cells depleted of ARL4D. This finding indicates that the phenotype was caused by the presence of GTP-binding-defective ARL4D rather than the absence of wild-type ARL4D protein. Further studies investigating how ARL4D regulates mitochondrial morphology may provide novel insights into the functional relationship(s) between mitochondrial fission and fusion, mitochondrial movement and transport, and cellular physiology.

The uncontrolled temporal and spatial binding of GTP or GDP to small GTPases can cause serious pathologies and disease. A mutation in the ARL6 Thr31 residue, which is important for GTP binding, was reported to cause Bardet-Biedl syndrome (BBS) [36]. We demonstrated here that GTP-binding-defective ARL4D is recruited to the mitochondria, where it affects mitochondrial morphology and membrane potential. Understanding whether GDP-bound ARL4D has physiological or pathological importance for mitochondrial dysfunction may be important. The upstream signaling pathways that control the localization of ARL4D and the detailed molecular mechanisms that are involved in the ARL4D-mediated maintenance of mitochondrial functions will be identified in further studies.

## Materials and Methods

### Antibodies

The rabbit anti-ARL4D [10] and anti-ARL4A [9] antibodies used have been described previously. The rabbit anti-ARL4C antibodies were raised against a peptide (PKSLPVAEIEKQLALH) that corresponds to amino acids 132–147 of ARL4C. Mouse monoclonal antibodies were used to detect myc 9E10 (BAbCO, Richmond, CA, USA), β-COP, α-tubulin (Sigma-Aldrich, St. Louis, MO, USA), Na^+^/K^+^ ATPase, Bax (Santa Cruz Biotechnology, Santa Cruz, CA, USA), VDAC (Cell Signaling Technology, Danvers, MA, USA), cytochrome c, EEA1, Tim23, Lamp-1, Bcl2, calnexin (BD Bioscience, San Jose, CA, USA), PARP (Millipore, Billerica, MA, USA), mannose 6-phosphate receptor (Calbiochem, La Jolla, CA, USA), pyruvate dehydrogenase and cytochrome c oxidase complex IV (Invitrogen, Carlsbad, CA, USA). The horseradish peroxidase-conjugated sheep anti-rabbit or anti-mouse immunoglobulin (IgG) antibodies were purchased from GE Healthcare (Little Chalfont, Buckinghamshire, United Kingdom), and the Alexa Fluor 594 or 488-conjugated anti-rabbit and anti-mouse antibodies were obtained from Invitrogen.

### Expression Plasmids

The cDNAs corresponding to ARL4D, ARL4C, ARL4A and their various mutants were cloned into the mammalian expression vector pSG5 (Stratagene, La Jolla, CA, USA), as previously described. These constructs were used to express untagged ARL4s [10]. To express C-terminally myc-tagged ARL4D(T35N), the ARL4D(T35N) fragments were cloned into pcDNA 3.1/myc-His A (Invitrogen). To express N-terminal His-tagged ARL4D(T35N) and (T52N), ARL4D(T35N) and ARL4D(T52N) fragments were cloned into pET32a (Novagen). The plasmid expressing Bax-GFP was kindly provided by Dr. Pei-Hsin Huang (National Taiwan University).

### Cell Culture and Transfection

COS-7 cells and HeLa cells were obtained from the American Type Culture Collection (ATCC) (Manassas, VA). Human rhabdomyosarcoma (RD) (ATCC No. CCL-136) cells were a kind gift from Dr. Passion Huang (National Taiwan University, Taipei, Taiwan); the original cell lines were obtained from the ATCC. COS-7 cells, HeLa cells, and RD cells were maintained in DMEM (Hyclone, Logan, UT, USA) with 10% FBS (Hyclone) in a humidified incubator containing 5% CO_2_ at 37°C. The cells were transiently transfected using the calcium phosphate method or by using Lipofectin or Lipofectamine 2000 (Invitrogen) according to the manufacturer’s protocols. The cells were harvested for analysis at 24–48 h after transfection.

### SDS-polyacrylamide Gel Electrophoresis (PAGE) and Immunoblotting Analysis

SDS-PAGE was performed according to the Laemmli method [37]. After electrophoresis, the proteins were transferred onto PVDF membranes (Millipore). The immunoblotting procedure was performed as previously described [10].

### Immunofluorescence Microscopy

Cells were processed for indirect immunofluorescence as previously described [10]. For the transferrin internalization assay, the cells were pre-incubated in serum-free medium containing 1% bovine serum albumin (BSA) for 1 h at 37°C and then incubated in the same medium supplemented with 50 µg/ml of Alexa 488-conjugated human transferrin (Invitrogen) for 30 min. The stained cells were examined with a Zeiss Axioplan 2 fluorescent microscope (Carl Zeiss MicroImaging, Inc, Oberkochen, Germany) with a 40×/1.3 NA oil objective lens. The images were acquired with a CCD camera (AxioCam HRm, Carl Zeiss) that was operated by the Axiovision 4.6 imaging software (Carl Zeiss). Some slides were optically sectioned, and out-of-focus signal was removed using the ApoTome system (Carl Zeiss). For confocal microscopy, the cells were inspected and imaged by a Nikon C1 confocal microscope with a 60×/1.4 NA oil objective lens and 488 nm or 543 nm lasers. The pinholes were set to scan layers <1 µm at a resolution of 1024×1024 pixels. The figures were assembled and labeled using Adobe Photoshop (Adobe Systems, Mountain View, CA). The quantitative localization analyses were performed by counting 100–200 randomly selected cells in each experiment.

### Subcellular Fractionation

The subcellular fractionation by differential centrifugation and density gradient centrifugation of the cellular postnuclear supernatant (PNS) was performed as previously described [10], [38]. Briefly, the cells were harvested by scraping in homogenization buffer (0.25 M sucrose, 10 mM Tris-Cl, pH 7.4, 1 mM magnesium acetate). The cells were then incubated on ice for 15 min, broken by passage through a 26-gauge needle (approximately 35 times) to approximately 70% complete breakage, and centrifuged (800× *g*) for 6 min at 4°C to remove the nuclei and unbroken cells. Samples (0.6 ml) of the PNS were loaded on top of iodixanol step gradients containing 2%, 6%, 10%, 14%, 18%, 22% and 26% iodixanol (0.6 ml each, total volume 4.2 ml) according to the manufacturer’s protocol (Axis Shield, Roskilde, Denmark; protocol S36). After centrifugation (100,000×*g*, 4°C, 3 h), 14 fractions (collected from the top) were concentrated by TCA precipitation, resuspended in SDS sample buffer, separated by SDS-PAGE, and analyzed by Western blotting. The protein levels in each fraction were quantified by densitometry and reported as the percentage of total protein recovered.

### Mitochondrial Fractionation and Proteinase K Sensitivity Assays

The cells were homogenized and subjected to subfractionation as previously described [31], [39]. Briefly, the cells were washed twice with ice-cold PBS, harvested in buffer containing 20 mM HEPES-KOH, pH 7.5, 10 mM KCl, 1.5 mM MgCl_2_, 1 mM EDTA, 1 mM EGTA, 2 mM dithiothreitol, 0.1 mM phenylmethylsulfonyl fluoride, 250 mM sucrose, and a protease inhibitor cocktail. The cells were homogenized at 4°C by passing the buffer through a 26-gauge needle 30 times. The homogenate was centrifuged to separate the PNS (1000×*g* for 5 min). The PNS fraction was further centrifuged at 10,000×*g* for 15 min, isolating the crude mitochondrial pellet. The crude mitochondrial pellet was washed once with isotonic mitochondrial buffer (20 mM Tris-Cl, pH 7.5, 0.07 M sucrose, 0.21 M mannitol, 2.5 mM EDTA, 2.5 mM EGTA) and resuspended in the same buffer. To determine the nature of the ARL4D-membrane association, the mitochondria-enriched fractions were treated with 0.1 M Na_2_CO_3_ (pH 12), 0.1% or 1% Triton X-100, or isotonic mitochondrial buffer (Mock) for 1 h on ice and centrifuged at 20,000×*g* for 20 min. The resulting pellet and supernatant fractions were analyzed.

The submitochondrial fractionation with digitonin was performed as previously described, with minor modifications [31]. The mitochondrial preparations (above) were incubated with increasing concentrations of digitonin (Sigma) in the isotonic buffer at the protein concentrations described in the figure legends. The preparations were incubated on ice for 30 min with occasional mixing by inversion. The preparations were then quickly diluted with 3 volumes of mitochondrial isotonic buffer and centrifuged at 20,000×*g* for 20 min at 4°C. The supernatant was saved separately. The pellet suspensions were assayed by Western blotting to detect marker protein release from each mitochondrial subcompartment. Mitochondrial preparations that were subjected to the same treatment in the absence of digitonin served as controls.

To isolate mitochondria for the proteinase K sensitivity assays, cells were homogenized in ice-cold cell homogenization medium (150 mM MgCl_2_, 10 mM KCl, 10 mM Tris-Cl, and 0.25 M sucrose, pH 6.7) with a Dounce homogenizer. The homogenate was then centrifuged (1000×*g*, 5 min, 4°C) to remove the nuclei and unbroken cells. The crude mitochondrial pellets resulting from the centrifugation (5000×*g*, 10 min, 4°C) of the PNS fraction were washed once with ice-cold sucrose/Mg^2+^ medium (0.15 M MgCl_2_, 0.25 M sucrose, and 10 mM Tris-Cl, pH 6.7), resuspended in ice-cold suspension medium (0.25 M sucrose and 10 mM Tris base, pH 7.0), and treated with proteinase K (50 µg/ml) plus or minus 0.1% Triton X-100 for 30 min on ice. The reactions were stopped by the addition of trichloroacetic acid (TCA, 10%). The proteins that were precipitated by TCA (15,000×*g*, 30 min, 4°C) were washed once with acetone and subjected to immunoblotting.

### Immunogold EM Labeling

The COS-7 cells that were transfected with ARL4D(T35N)-myc were fixed with 0.1% glutaraldehyde and 4% formaldehyde in sodium phosphate buffer (pH 7.2). Cryosectioning and electron microscopy were performed as previously described [40].

### Measurement of Mitochondrial Membrane Potential (ΔΨm)

The ΔΨ_m_ was measured by immunostaining using MitoTracker Red (Invitrogen) or by flow cytometry using JC-1 (Invitrogen). For MitoTracker Red staining, the cells were incubated with prewarmed (37°C) medium containing 100 nM MitoTracker Red CMXRos or 300 nM CM-H2XRos (Invitrogen) for 30 min at 37°C, washed twice with prewarmed medium to remove excess dye, and then fixed in prewarmed growth medium containing 4% formaldehyde for 15 min at 37°C. The quantification of MitoTracker fluorescence was performed using Axiovision 4.6 software (Carl Zeiss). The images were taken under the same conditions, and any intensity-saturated images were excluded from the analysis. The area around each cell was delineated, and the fluorescence intensity was measured in pixels. The background fluorescence was obtained from a cell-free field in each image and subtracted from the actual fluorescence. The cationic dye JC-1 emits a bright red fluorescence after forming aggregates in intact mitochondria. Mitochondrial aggregates do not form when the ΔΨ_m_ is disrupted; rather, the dye remains in monomeric form in the cytoplasm and emits green fluorescence. The cells were incubated with 2 µM JC-1 in medium for 30 min at 37°C and harvested by trypsinization. After washing the cells with PBS twice, the green (λ_em_ = 527 nm) and red (λ_em_ = 590 nm) fluorescence was measured using a FACSCalibur flow cytometer (BD Bioscience) and CellQuest software. The results are presented as the mean ratio of red to green fluorescence intensity. The raw flow cytometry data for the JC-1 red-to-green ratio, representing the mean intensities of 10^4^ cells, included cells that had not been transfected or in which ARL4D was not localized to the mitochondria. Therefore, we calculated the ΔΨ_m_ of the mitochondrial ARL4D(T35N)-and ARL4D(T35NΔ16C)-expressing cells by taking the transfection efficiency (∼40% for each plasmid) and the percentage of the different ARL4D mutants localized to the mitochondria (∼60% for ARL4D(T35N) and ∼95% for ARL4D(T35NΔ16C)) into account.

### Generation of ARL4D-depleted Stable Transfectants

The ARL4D gene was silenced using synthetic 21-nt RNA duplexes according to previously described protocols [10]. The following pairs of oligonucleotides with hairpin, terminator, and overhanging sequences were annealed and inserted into the BglII–HindIII sites of the pSUPER or pSUPER.gfp/neo vectors (OligoEngine, Seattle, WA, USA) to generate vectors expressing short-hairpin RNAs (shRNAs) targeting human ARL4D: 5′-gatccccggtggagttgcaccgaatcttcaagagagattcggtgcaactccacctttttggaaa-3′ and 5′-agcttttccaaaaaggtggagttgcaccgaatctctcttgaagattcggtgcaactccaccggg-3′. The gene-specific insert for these primers specifies a 19-nucleotide sequence corresponding to nucleotides 339–357 immediately downstream of the ARL4D transcriptional start site. The pSUPER plasmids were transfected directly into the cells. The cells were harvested for immunoblotting and immunofluorescence at 48 h after transfection. To select for stable transfectants, the ARL4D shRNA/pSUPER.gfp/neo-transfected or pSUPER.gfp/neo-transfected HeLa cells were grown in medium containing 500 µg/ml G418 (Invitrogen). ARL4D expression was then confirmed by Western blot.

### Cell Viability and Colony Formation Assays

Cell viability was determined using the MTT [3-(4,5-dimethylthiazol-2-yl)-2,5-diphenyltetrazolium bromide] assay. In brief, the cells were plated at 2×10^4^ cells/well in 96-well plates 24 h after transfection and incubated in medium (100 µl) for 24 or 48 h. After incubation with 100 µl of medium containing 0.5 mg/ml MTT for an additional 3 h at 37°C, the accumulated formazan was dissolved in 100 µl of DMSO, and the absorbance of this solution was measured at 570 nm. For the colony formation assay, equal numbers of transfected cells were seeded in 100-mm culture dishes and cultured under G418 selection (800 µg/ml). After two weeks, the colonies were stained with 0.1% crystal violet in 50% methanol and photographed.

### *In vitro* Guanine Nucleotide Binding Assay

Preparation and purification of recombinant proteins were carried out as described previously [41], [42]. Nucleotide binding assays were carried out in 150 µl reaction mixtures with 100 mM His-tagged ARL4D(T35N) or His-tagged ARL4D(T52N) and 1 µM mant-GDP or mant-GTP (Molecular Probes) in a buffer containing 20 mM Tris-Cl, pH 8, 100 mM NaCl, 1 mM MgCl_2_, and 5 mM DTT. The mixtures were incubated at 4°C for 16 h and transferred into a 96-well black plate. Fluorescence was evaluated using a SpectraMax M5 (Molecular Devices) spectrofluorimeter excited at 355 nm and emissions were monitored between 400 and 600 nm and fluorescence intensities were measured at 448 nm.

## Supporting Information

Figure S1 **ARL4D(T35N) localizes to the mitochondria.** COS-7 cells were transfected with a plasmid encoding untagged ARL4D(T35N) and incubated with antibodies against ARL4D, βCOP, calnexin, EEA1, transferrin, mannose 6-phosphate receptor (M6PR) or Lamp-1. The cells were then examined by confocal microscopy. The nuclei were stained with Hoechst 33258. Bar, 10 µm.
(PDF)

Figure S2 **Localization of the mutant ARL4 family proteins and their effects on mitochondrial membrane potential.** (A) COS-7 cells were transfected with plasmids encoding untagged ARL4D(T52N), ARL4A(T34N), or ARL4C(T27N) and incubated with antibodies against ARL4D, ARL4A, or ARL4C plus cytochrome c, as indicated. (B) COS-7 cells were transfected with plasmids encoding untagged ARL4D(T52N), ARL4A(T34N), or ARL4C(T27N) for 48 h and stained with MitoTracker CMXRos before fixation and immunostaining with antibodies against ARL4D, ARL4A, or ARL4C. Bars, 10 µm.
(PDF)

Figure S3 **T35N and T52N mutants of ARL4D bind to N-methylanthraniloyl-GDP (mGDP).** Fluorescence intensity of mGTP or mGDP was measured (λex = 355 nm, λem = 400–600 nm) in the absence or after 16 h incubation with (A) ARL4D(T35N) or (B) ARL4D (T52N) at 4°C. Green and purple curves indicate fluorescence intensity from mGDP alone or mGDP-ARL4D complexes. Dark blue and red curves indicate fluorescence intensity from mGTP alone or mGTP-ARL4D complexes. ARL4D(T35N)-mGDP and ARL4D(T35N)-mGTP showed slight increase (3x and 1.3x, respectively) in fluorescence intensity compare to nucleotides alone. ARL4D(T52N)-mGDP complex showed a sixfold increase in fluorescence intensity while ARL4D(T52N)-mGTP complex showed no increase. Guanine nucleotide binding assay was carried out as described in Materials and Methods.
(PDF)
