# Supplementary figures and images for: GTP-Binding-Defective ARL4D Alters Mitochondrial Morphology and Membrane Potential
Source: PLoS One. 2012 Aug 21;7(8):e43552. doi: 10.1371/journal.pone.0043552 (PMC3424131; doi:10.1371/journal.pone.0043552)

ARL4D(T35N)

H33258

$\beta$ COP

Calnexin

EEA1

Transferrin

M6PR

Lamp-1

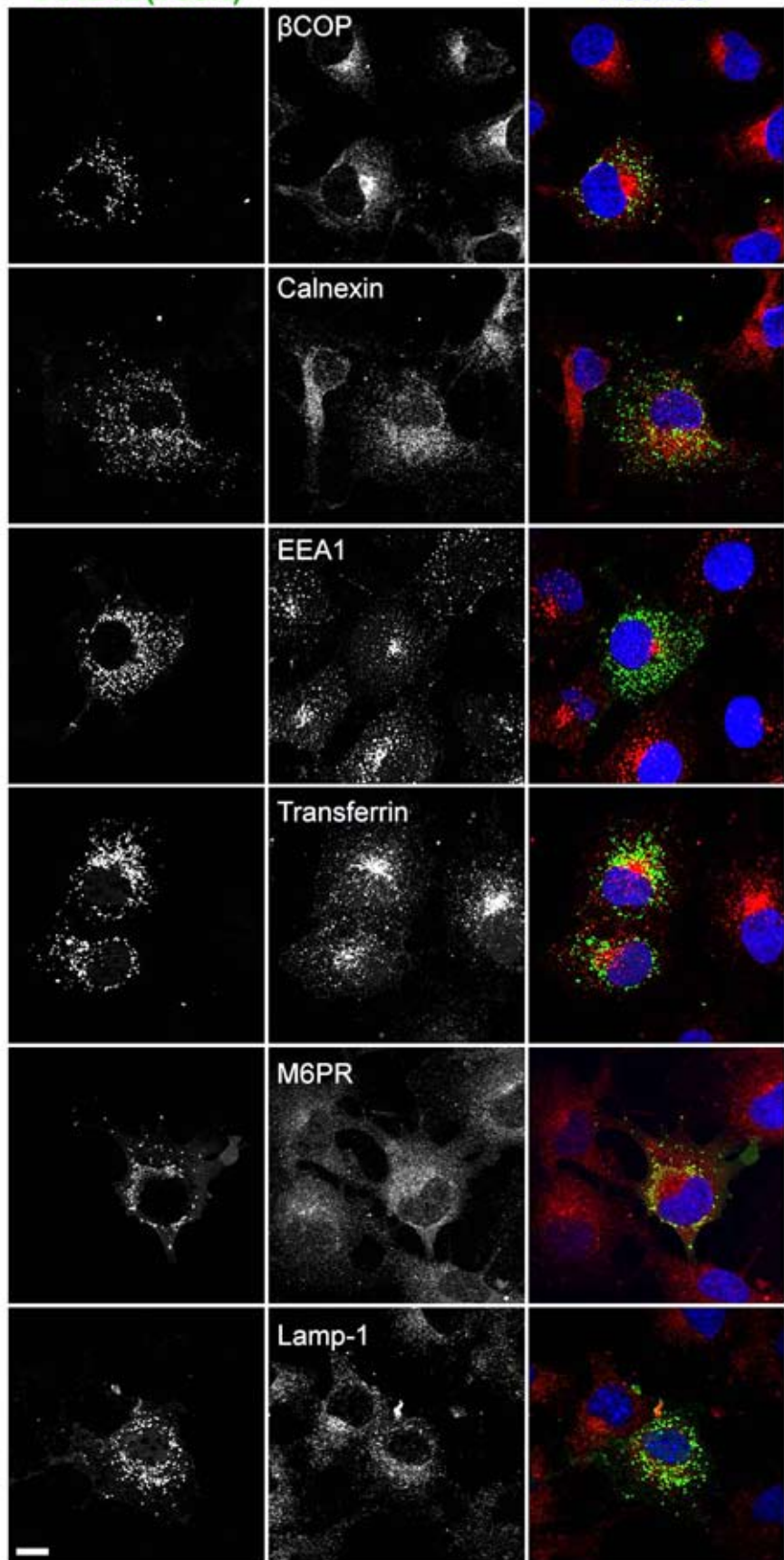

Supplement: Figure S1 — ARL4D(T35N) localizes to the mitochondria. COS-7 cells were transfected with a plasmid encoding untagged ARL4D(T35N) and incubated with antibodies against ARL4D, βCOP, calnexin, EEA1, transferrin, mannose 6-phosphate receptor (M6PR) or Lamp-1. The cells were then examined by confocal microscopy. The nuclei were stained with Hoechst 33258. Bar, 10 µm. (PDF) [file pone.0043552.s001.pdf]

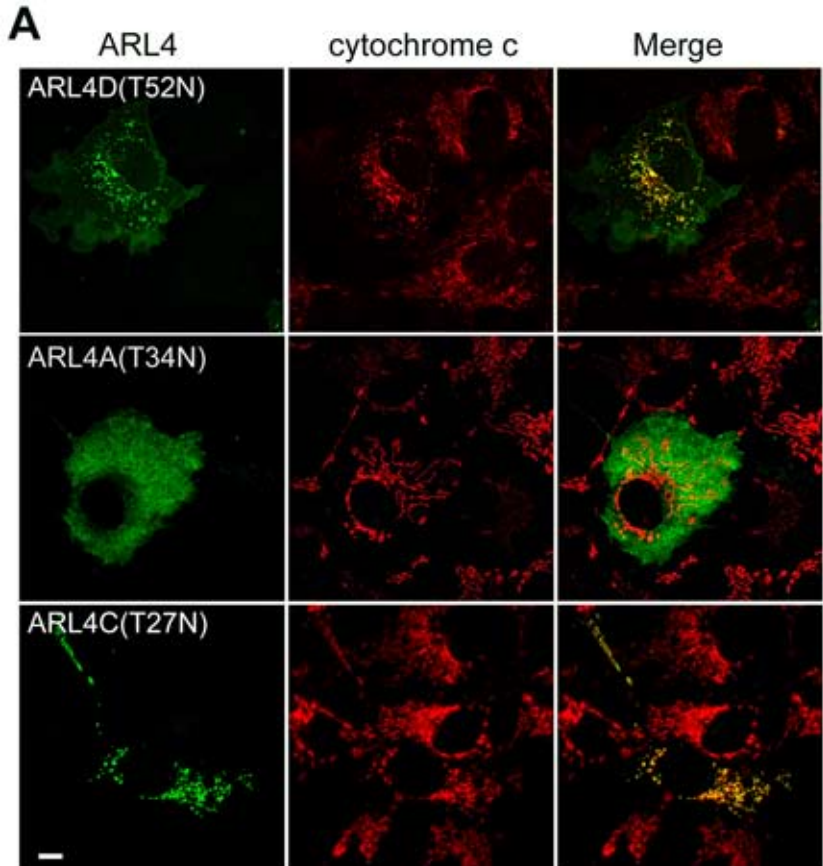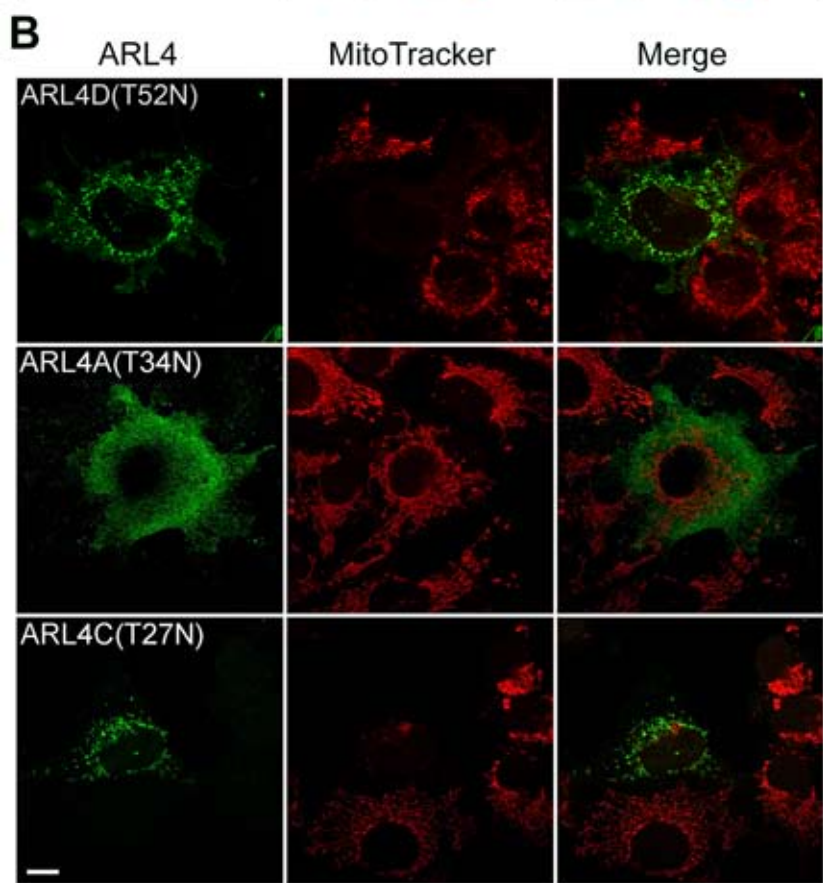

Supplement: Figure S2 — Localization of the mutant ARL4 family proteins and their effects on mitochondrial membrane potential. (A) COS-7 cells were transfected with plasmids encoding untagged ARL4D(T52N), ARL4A(T34N), or ARL4C(T27N) and incubated with antibodies against ARL4D, ARL4A, or ARL4C plus cytochrome c, as indicated. (B) COS-7 cells were transfected with plasmids encoding untagged ARL4D(T52N), ARL4A(T34N), or ARL4C(T27N) for 48 h and stained with MitoTracker CMXRos before fixation and immunostaining with antibodies against ARL4D, ARL4A, or ARL4C. Bars, 10 µm. (PDF) [file pone.0043552.s002.pdf]

**A**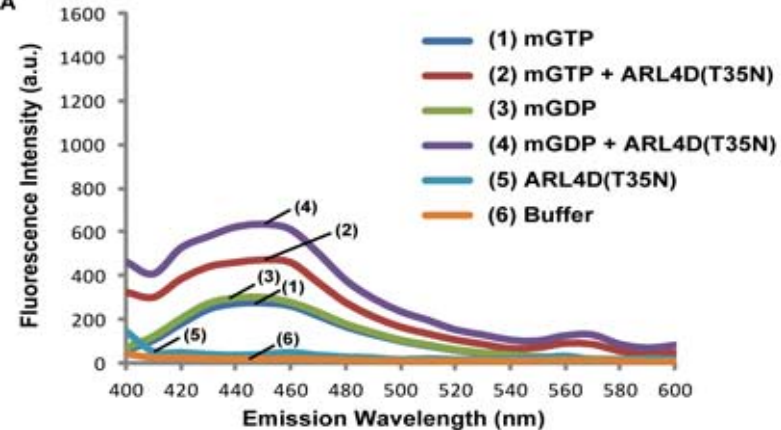**B**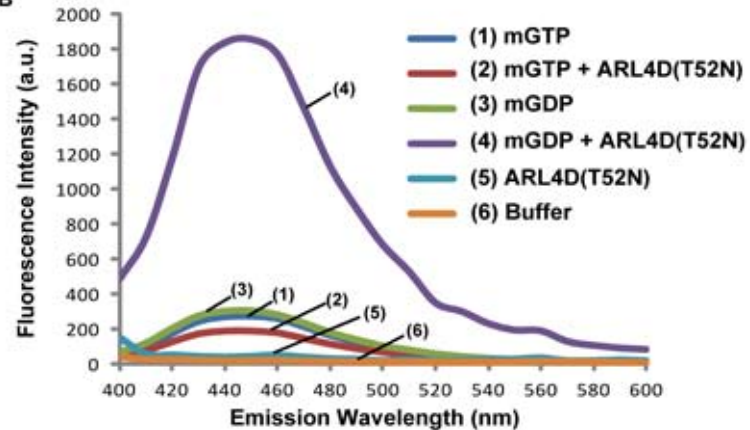

Supplement: Figure S3 — T35N and T52N mutants of ARL4D bind to N-methylanthraniloyl-GDP (mGDP). Fluorescence intensity of mGTP or mGDP was measured (λex = 355 nm, λem = 400–600 nm) in the absence or after 16 h incubation with (A) ARL4D(T35N) or (B) ARL4D (T52N) at 4°C. Green and purple curves indicate fluorescence intensity from mGDP alone or mGDP-ARL4D complexes. Dark blue and red curves indicate fluorescence intensity from mGTP alone or mGTP-ARL4D complexes. ARL4D(T35N)-mGDP and ARL4D(T35N)-mGTP showed slight increase (3x and 1.3x, respectively) in fluorescence intensity compare to nucleotides alone. ARL4D(T52N)-mGDP complex showed a sixfold increase in fluorescence intensity while ARL4D(T52N)-mGTP complex showed no increase. Guanine nucleotide binding assay was carried out as described in Materials and Methods. (PDF) [file pone.0043552.s003.pdf]
